# Assessing Drug Target Association Using Semantic Linked Data

**DOI:** 10.1371/journal.pcbi.1002574

**Published:** 2012-07-05

**Authors:** Bin Chen, Ying Ding, David J. Wild

**Affiliations:** 1 School of Informatics and Computing, Indiana University, Bloomington, Indiana, United States of America; 2 School of Information Science and Library, Indiana University, Bloomington, Indiana, United States of America; University of North Carolina, United States of America

## Abstract

The rapidly increasing amount of public data in chemistry and biology provides new opportunities for large-scale data mining for drug discovery. Systematic integration of these heterogeneous sets and provision of algorithms to data mine the integrated sets would permit investigation of complex mechanisms of action of drugs. In this work we integrated and annotated data from public datasets relating to drugs, chemical compounds, protein targets, diseases, side effects and pathways, building a semantic linked network consisting of over 290,000 nodes and 720,000 edges. We developed a statistical model to assess the association of drug target pairs based on their relation with other linked objects. Validation experiments demonstrate the model can correctly identify known direct drug target pairs with high precision. Indirect drug target pairs (for example drugs which change gene expression level) are also identified but not as strongly as direct pairs. We further calculated the association scores for 157 drugs from 10 disease areas against 1683 human targets, and measured their similarity using a 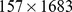 score matrix. The similarity network indicates that drugs from the same disease area tend to cluster together in ways that are not captured by structural similarity, with several potential new drug pairings being identified. This work thus provides a novel, validated alternative to existing drug target prediction algorithms. The web service is freely available at: http://chem2bio2rdf.org/slap.

## Author Summary

Modern drug discovery requires the understanding of chemogenomics, the complex interaction of chemical compounds and drugs with a wide variety of protein target and genes in the body. A large amount of data pertaining to such relationships exists in publicly-accessible datasets but it is siloed and thus impossible to use in an integrated fashion. In this work we have integrated and semantically annotated a large amount of public data from a wide range of databases, including compound-gene, drug-drug, protein-protein, drug-side effects and so on, to create a complex network of interactions relating to compounds and protein targets. We developed a statistical algorithm called Semantic Link Association Prediction (SLAP) for predicting “missing links” in this data network: i.e. compound-target interactions for which there is no experimental data but which are statistically probable given the other relationships that exist in this set. We present validation experiments which show this method works with a high degree of accuracy, and also demonstrate how it can be used to create a drug similarity network to make predictions of new indications for existing drugs.

## Introduction

Understanding the interaction of drugs with multiple targets can identify potential side effects and toxicities [1]–[3], as well as possible new applications of existing drugs [4]–[8]. Many efforts have been made to integrate drug-target interactions in a large scale [9]–[12]. A variety of computational approaches have been previously explored for predicting drug-target interactions, including molecular docking [3], [13], [14], ligand-based predictive models [15], [16], phenotype similarity (side effect similarity [17] or gene expression profile similarity [18]) and chemical ontology similarity [19]. Some similarity measurements have been combined to elucidate drug targets [20]. Network analysis based on the topology of known drug target network has also been utilized for drug target prediction, but is currently limited to small data sets [21], [22].

Recent advances in the Semantic Web [23] have enabled the creation of large heterogeneous networks of experimental and other data in life sciences (for example: Chem2Bio2RDF [24], LODD [25], Bio2RDF [26], OpenPHACTS (http://openphacts.org), linked life data (http://linkedlifedata.com) and Linked Open Data (http://linkeddata.org)), where the nodes can include physical and abstract entities (compounds, protein targets, substructures, side effects, diseases, pathways, tissues, gene ontology terms and so on), and the edges (or links) represent various relations between objects such as drug-drug interactions, and drug target interactions, protein-protein interactions and so on. The ability to easily integrate heterogeneous datasets in a meaningful fashion makes semantic technologies attractive, although it is only recently that supporting technologies have adequately matured to make them useful in the biological sciences: in particular the advent of fast triple stores for data storage, the SPARQL query language (http://www.w3.org/TR/rdf-sparql-query/) for searching, and the OWL ontology language (http://www.w3.org/TR/owl-features/) for the description of ontologies. Despite remaining deficiencies which are being addressed in the Semantic Web community (including difficulty weighting edges and maintaining provenance information) there are now many examples of successful use of semantics in the life sciences [27]. In contrast to hyperlinked data, semantic linked data encodes explicit meanings of nodes and links, allowing traversing from one node to another via particular kinds of relationship. Prediction of links not in the dataset, based on the existing links, is widely used in social networking, in which it is assumed that two nodes are similar if they share similar topology (e.g., a certain number of neighbors, and similar shortest paths) [28]–[30]. For example, in a coauthorship network, two authors are similar in terms of research interests if they coauthor lots of papers, hence their potential collaboration could be predicted (it should be noted that social networks generally only deal with positive relationships; drug discovery data is different in that negative relationships such as inactivity are important).

In this work, we sought to use such semantic methods to integrate and annotate the data in relation to drug target interaction, constructing a heterogeneous network composed by over 290 k nodes and 720 k edges. We further developed a statistical model called Semantic Link Association Prediction (SLAP) to assess the association of drug target pairs and to predict missing links. An association score is calculated based on the topology and semantics of the neighborhood. We demonstrate that SLAP can correctly identify known drug target pairs from random pairs with high accuracy and can also identify indirect drug target relations (e.g., the change of gene expression level). The association scores of a drug against a set of targets constitute a biological signature that allows assessing the similarity of drugs in the context of the whole system. The resulting drug similarity network clusters drugs from the same therapeutic indication in ways not observed using chemical structure similarity, and can also be used to identify potential new indications for existing drugs.

## Results

### Semantic linked data

**Figure 1:**
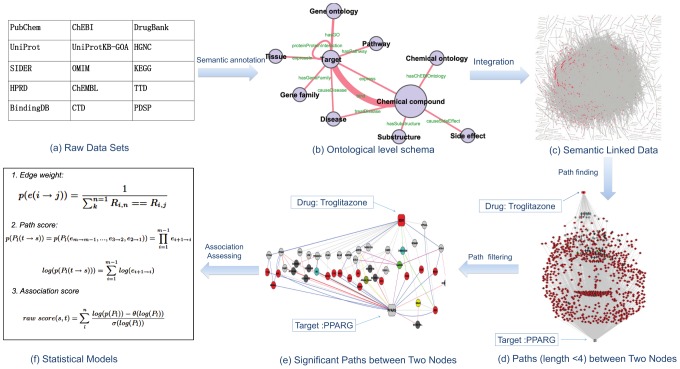
SLAP pipeline. An ontology is used to annotate public data sets and integrate them into a semantic linked network. Two nodes are linked by one or more number of paths, but only a small number of significant paths are kept for association estimation. The path significance and drug target associations are assessed by statistical models derived from random samples.

**Figure 2:**
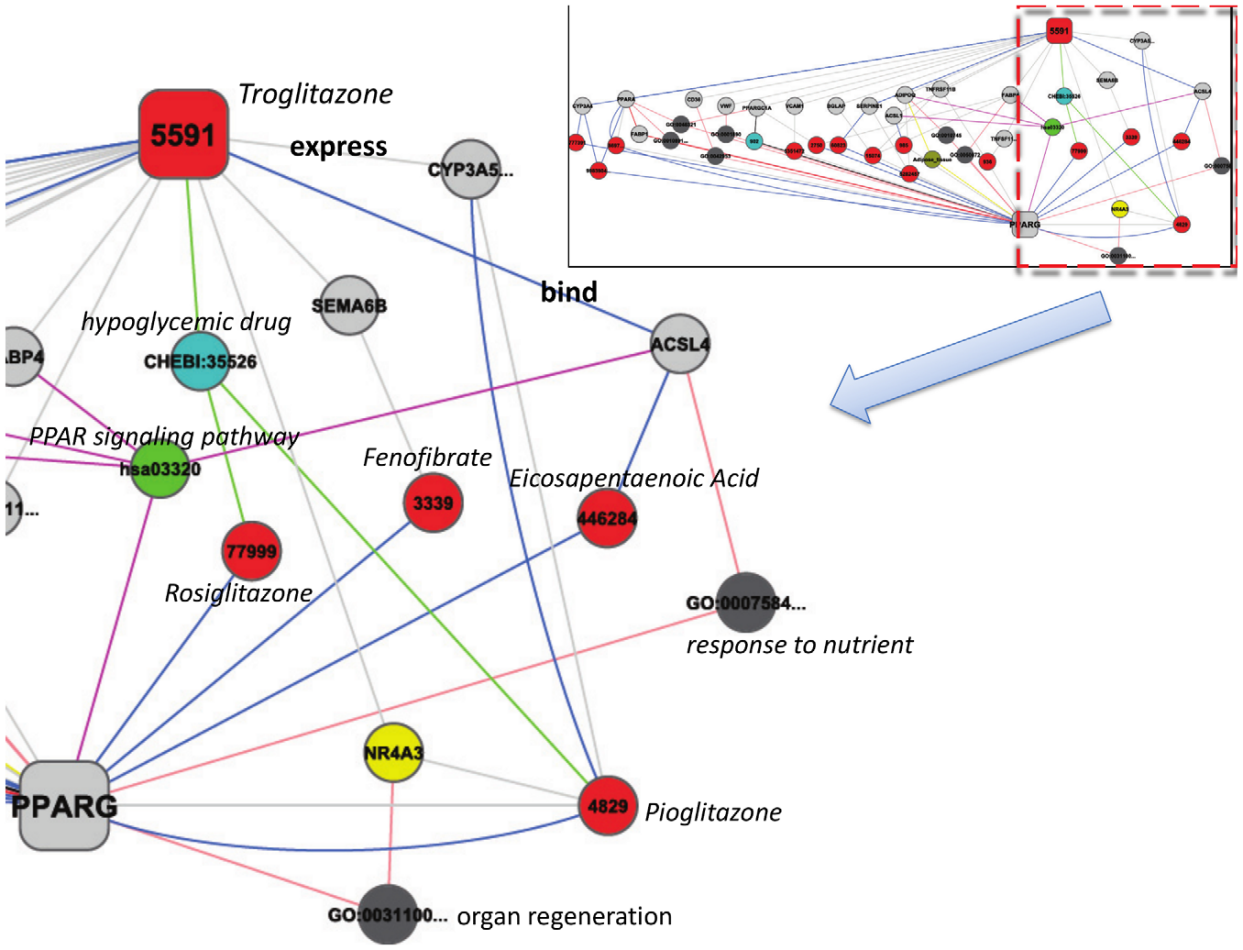
Paths between Troglitazone (label as PubChem ID: 5591) and PPARG with length 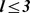. The nodes and edges are colored by their classes and edge types respectively. Some nodes are annotated additionally to help understand.

The SLAP pipeline is shown in Figure 1. A heterogeneous network consisting of 295,897 nodes and 727,997 edges was constructed from 17 public data sources pertaining to drug target interaction. Every node and edge was semantically annotated using a systems chemical biology/chemogenomics ontology previously developed in our labs [31]. The nodes were grouped into 10 classes which are linked by 12 types (Figure 1b). A single node is an instance of a corresponding class, for example: a node for the drug Troglitazone (labeled as 5591 in Figure 2) is an instance of class Chemical Compound. We term paths of nodes and edges that share the same semantics (but different data) path patterns - each path is an instance of a path pattern. Table 1 shows 6 path pattern examples between Drugs and Targets. In Figure 2, the path from node 5591 (Troglitazone) to node PPARG (Glitazone receptor) via ACSL4 (Long-chain-fatty-acid CoA ligase 4) and 446284 (Eicosapentaenoic acid) is an instance of the path pattern 1 in Table 1. We can interpret this path as indicating Troglitazone could bind to ACSL4 which shares compound Eicosapentaenoic acid with target PPARG. With the assumption that two nodes are associated if they link to at least one other node, or their linked nodes are linked, their relations can be assessed by the analysis of the links (or paths) between the two nodes [32]. The strength of their relation in the network can be measured by the distance, the number of shortest paths and other topological properties between the two nodes. In our example of the relationship between Troglitazone and target PPARG, several paths provide “evidence” of a relationship: Troglitazone and Rosiglitazone both are hypoglycemic drugs and the latter is the ligand of PPARG; Troglitazone binds to ACSL4 which shares pathway(PPAR signaling pathway), ligand (Eicosapentaenoic acid) and GO term (response to nutrient) with PPARG. A total of 1684 paths (length 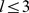) belonging to 10 path patterns contribute to their relation.

**Table 1:** Path pattern examples.

Path patterns	AUROC
Chemical/Drug–*bind*–Target–*bind*–Chemical/Drug–*bind*–Target	0.850
Chemical/Drug–*bind*–Target–*hasGo*–GO–*hasGO*–Target	0.824
Chemical/Drug–*hasSubstructure*–SubStructure–*hasSubstructure*–Chemical/Drug–*bind*–Target	0.620
Chemical/Drug–*express*–Target–*hasPathway*–Pathway–*hasPathway*–Target	0.495
Chemical/Drug–*express*–Target–*hasTissue*–Tissue–*hasTissue*–Target	0.501
Chemical/Drug–*express*–Target–*PPI*–Target	0.501

Edge types are presented as italic. AUROC shows the performance of predicting drug target interaction with the pattern alone. The first three patterns are more informative than the last three in their capability to contribute to the associations.

### Pattern score distribution

Each path between two nodes may contribute to the relation between them, but the degree of contribution varies depending on path distance and the weight of the edges involved in the path. For example, a gene ontology molecular function term (GO:0005515) shared by proteins is not as informative as a binding term (GO:0005488) in assessing the similarity of two proteins. Thus the weight of the edge linking one protein node to the molecular function node is lower than that linking to the binding node. According to this observation, we developed a statistical model to measure the weight of edges as well as the significance of paths (see methods). The model takes into account the distance and the weight of each edge, and renders a raw score indicating the strength of each path. We found that the raw scores within the same path pattern are normally distributed, while the mean and standard deviation of patterns are different (Figure S1). Z scores converted from raw scores based on pattern score distribution are used to measure the contribution to the association: the higher the z score, the more contribution the path has. The sum of z scores of all paths is defined as association score indicating the association strength of the drug target pair. The logarithm of association scores of random drug target pairs fit to a normal distribution (Figure S2), that enables calculation of the significance of a given association score. For our Troglitazone & PPARG example, the p-value is 9.06E-6, indicating a strong association.

### Pattern importance

A low p-value between a drug-target pair indicates a strong probability of association between the drug and target, but it does not necessarily mean the drug and target would interact biologically. Some patterns may be uninformative. We therefore considered each pattern as a feature and assessed each feature alone for its ability to identify drug-target pairs from random pairs across the set. Table 1 lists three informative patterns and three uninformative patterns along with ROC scores. The first two patterns illustrate the drug likely interacts with a protein that shares commonalities in terms of GO or ligand binding profile with an existing target that the drug already is known to interact with. The third pattern indicates that the drug likely interacts with a protein with which another structural similar drug could interact. As a result of this analysis, 12 “uninformative” patterns were removed. The sum of z score of a given pair is the sum of z scores of the paths belonging to the informative patterns.

### Association scores of drug target pairs

**Figure 3:**
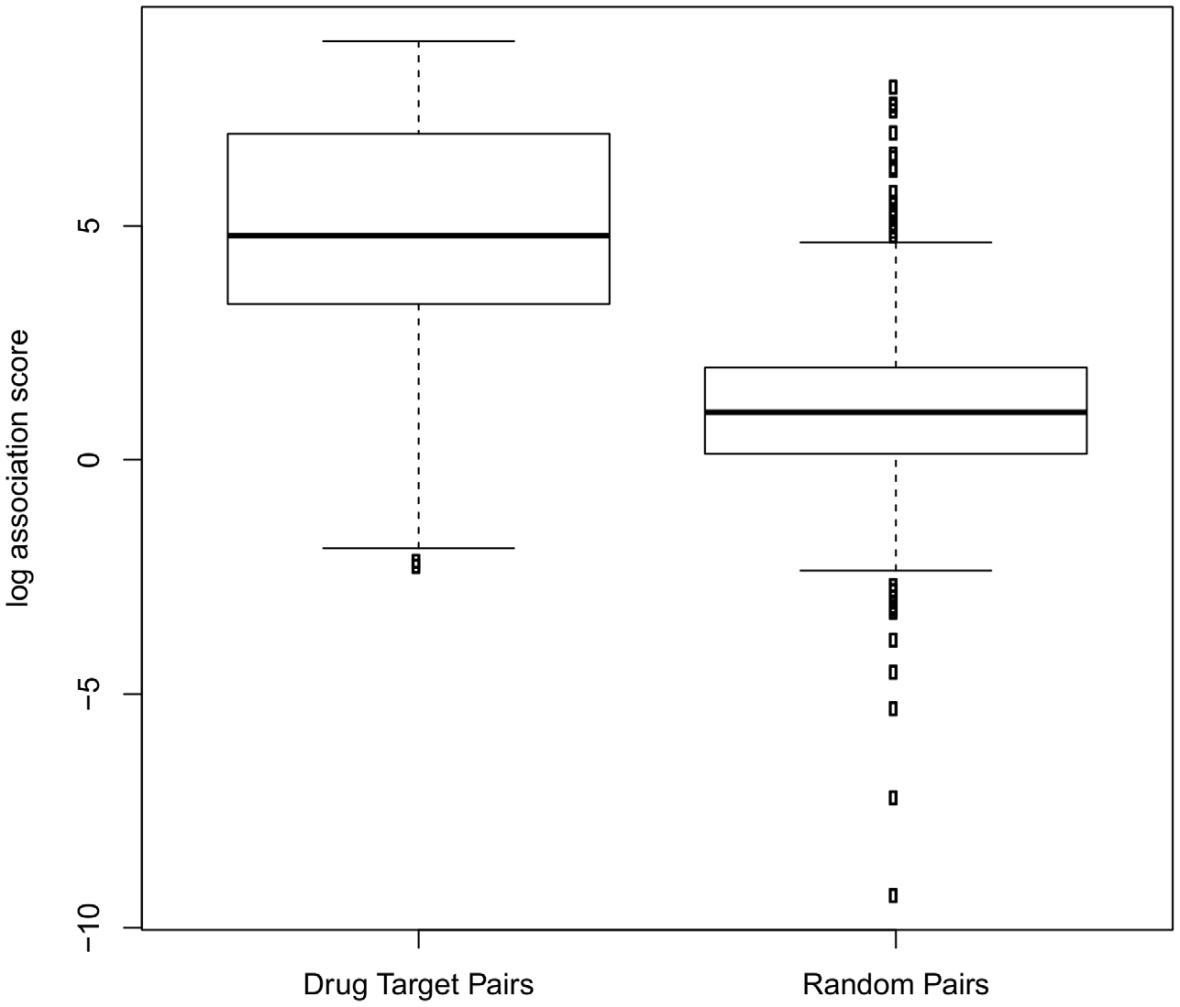
Logarithmic association score distribution of drug target pairs.

**Figure 4:**
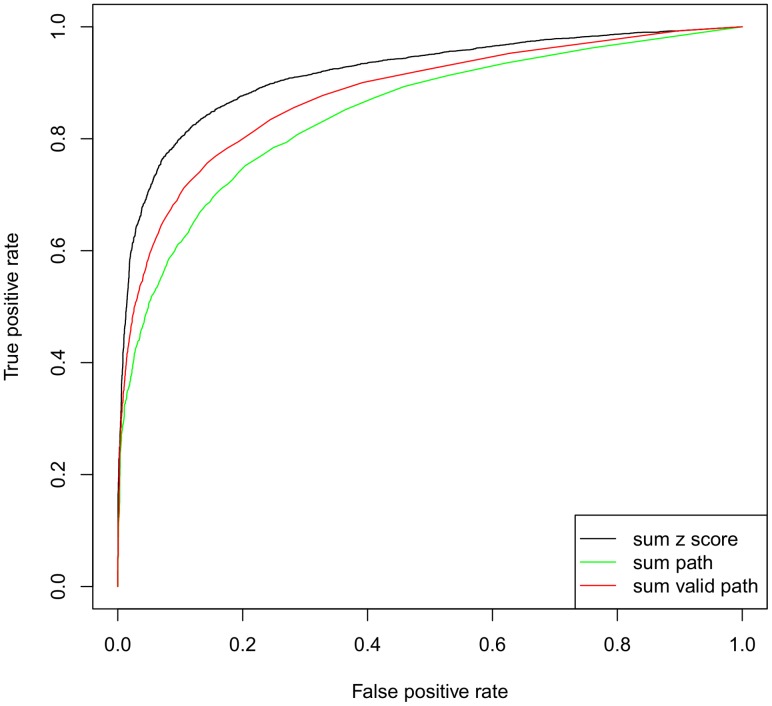
ROC curves among different prediction methods. Valid paths mean their 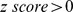.

We randomly selected 1000 known drug target pairs from DrugBank and compared their association scores with 1000 random pairs of drugs and targets sampled from DrugBank. For each drug target pair, their direct link was removed in the score calculation so that their association is only determined by their neighborhood properties. We thus aimed to test the ability of SLAP to correctly identify “missing links” in the data, with the assumption that this might be used, for instance, to profile a group of compounds against an identified set of targets. As Figure 3 shows, random pairs have a broad range of scores, but most of them are close to zero. Overall, real drug-target pairs have much higher scores than random pairs (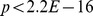 using paired t test). We also took all drug target pairs from DrugBank (in total 5607 pairs in which 4508 pairs have at least one path with length 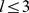). We sampled the same number of random drug target pairs as decoys to check the capability of identifying real drug target pairs by SLAP. We compared SLAP with other link prediction methods adopted in social network analysis [32]. The AUROC of SLAP is 0.92, outperforming other methods (i.e., the number of shortest paths, and the number of valid paths)(Figure 4). As the ratio between true drug target pairs versus random pairs decreases (e.g., ratio = 1/12), the ROC scores do not vary very much (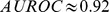) and SLAP still performs much better than others, although the precision goes down considerably (Figure S5). Even when random pairs are 12 times more than positive pairs, the precision still can reach 0.6 while recall is 0.7. In addition, we noticed using the sum (or max or mean) of raw score of the shortest path (without converting into z scores) performs as a random choice, indicating the importance of introducing random samples. Since several drug target prediction approaches reported that the performances may vary among different target classes [33], we grouped the drug target pairs into 5 classes (Enzyme, Membrane Receptor, Ion Channel, Transporter and Transcription Factor), and found that the score does not have any preference to a particular target class, indicating SLAP is capable of treating different classes of protein targets(Figure S4).

As far as we are aware, SLAP is the only large predictive network model that has been applied to drug discovery data. However other drug-target prediction methods have been the subject of recent publications [7], [17], [34], and we thus sought to consider how the effectiveness of SLAP compares with these methods. We ran SLAP against 23 drug target pairs (including 15 aminergic G-protein-coupled receptors and 8 cross-boundary targets) predicted and confirmed in using the SEA method [7], a novel drug prediction method based on similarity analysis. 9 pairs of aminergic GPCRs were identified by SLAP (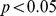); 1 pair was not decided (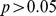); the rest of GPCRs have no mappings in the network (the drug was not found in the network), while only one of eight cross-boundary targets was identified by SLAP (see Table S4), indicating that, SLAP is not capable of finding surprising pairs (cross-boundary targets). For example, Vadilex, an ion channel drug was predicted in SEA as a ligand of a transporter, a totally different target, but was not identified by SLAP. Nevertheless, SLAP performs considerably well among GPCRs in this case.

In addition, we examined drug target pairs from MATADOR [35] which serves as an external dataset for validation. 1065 direct pairs were collected, of which 444 pairings are not represented in our network. 560 out of 621 known pairs and 170 out of 444 unknown drug target pairs were identified by SLAP (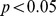).

### Comparison with Connectivity Maps

By calculating association scores across multiple targets, SLAP can be used to build a polypharmacology profile of a drug even when a full data matrix is not available from drug-target experiments. We took all the 164 small molecules from the Connectivity Map (CMap), an online dataset mapping relationships of disease profiles to known drugs [18], and 113 molecules that were mapped to our network were used to build a library. The association scores of these compounds against 1683 targets were calculated, yielding a 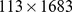 score matrix. The targets of which max score is smaller than 113 (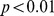) were eliminated so that each remaining protein is a target of at least one drug. After this filtering, a matrix composed by 113 compounds and 679 targets was built. We used the signature of a given drug to compare it with all the compounds in the library to find the most similar drugs according to Pearson correlation coefficient. Following the CMap approach, 8 queries including 2 HDAC inhibitors, 1 estrogen and 5 Phenothiazines were created and the similar pairs are listed in Table S5. We set 0.75 as threshold. 21 pairs were identified by SLAP, 19 out of 21 pairs were actually the pairs identified by CMap. SLAP recovered all HDAC inhibitors, but missed two hits (Genistein and Tamoxifen) for estrogen, however, both hits rank very high. Two Phenothiazines were not recovered using this similarity threshold, but they are quite similar to other three Phenothiazines compared to the remaining compounds in the library. The results show that most of hits identified by SLAP are true positive, indicating that the profiles derived from SLAP resemble gene expression profiles being used for target identification.

### Assessing drug similarity from biological function

We took 157 drugs from 10 disease areas to determine whether SLAP is able to distinguish drugs from different therapeutic areas. For each drug, we ran SLAP against 1683 human targets and got an association score for each drug target pair, creating a 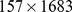 score matrix. We only kept the drugs and targets in which the max score is at least larger than 113 (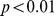) to make sure each drug has at least one valid target and each target has at least one valid drug. The matrix was then reduced to 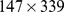, followed by the correlation calculation of every drug pairs. Only pairs with coefficient 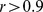 were taken to build a network (see methods).

#### Identifying mechanisms of action

**Figure 5:**
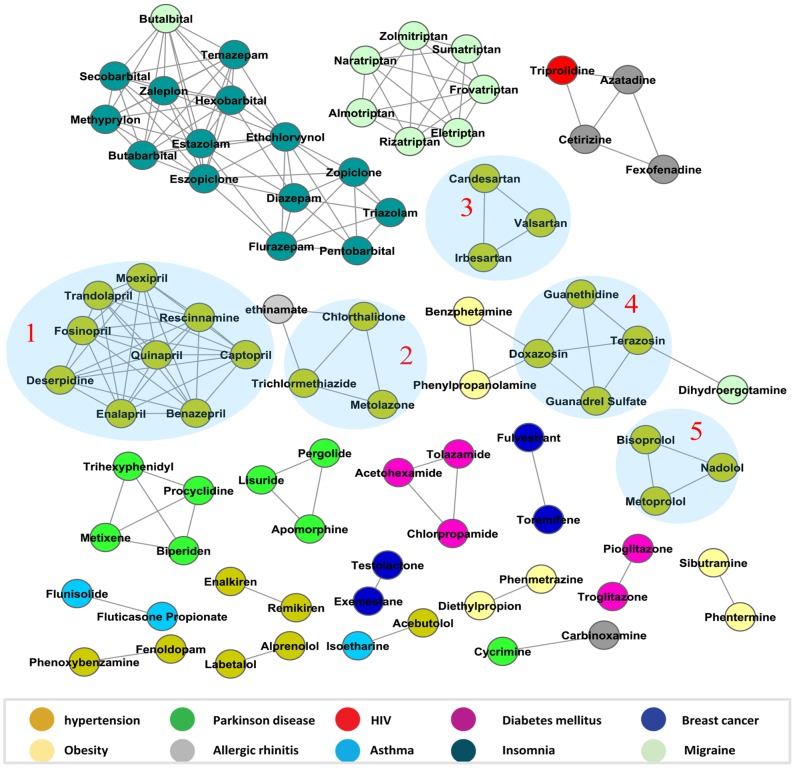
Drug similarity network. Each node presents a drug, and two nodes are linked if their similarity (in terms of polypharmacology profile) 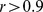. The drugs are colored by their therapeutic indication. Five hypertension related clusters are shadowed.

Drugs with the same therapeutic indication tend to cluster together (Figure 5), and we also found that these subcluster by mechanism of action. For example, hypertension drugs, subcluster into ACE inhibitors, thiazide-based diuretics, angiotensin II antagonists, alpha-adrenoreceptor antagonists and beta blockers (clusters 1–5 in Figure 5 respectively).

#### Calculating similarity of drugs by biological function

Mostly, chemically similar drugs have similar biological function. However, small changes of structure may also result in big change of function, or even totally different indications. For example, adding a methyl group to Levodopa, a dopaminergic agent for Parkinson's disease, makes it Methyldopa, an antiadrenergic (Tanimoto coefficient = 0.89; Figure S6b) for antihypertension. They are distinguished by SLAP (similarity 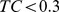). The antihypertensive effect of Methyldopa is likely due to its metabolism to alpha-methylnorepinephrine (CID:3917). SLAP is still able to distinguish its metabolite from Levodopa (similarity 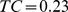). Conversely, biologically similar drugs identified by SLAP are not necessarily structural similar. For example, a number of drugs treating insomnia are quite different in term of structure(Figure S6a), but they are clustered together by SLAP.

#### Drug repurposing

Some drugs with very different indications are clustered together. This may suggest some new indications of drugs or possible side effect considerations. For example, Butalbital, a Barbiturate used to treat Migraines, is clustered with nine Insomnia drugs, two of which (Butibarbital and SecoBarbital) are Barbiturates. Barbiturates act as central nervous system depressants, capable of producing all levels of CNS mood alteration including Insomnia. Triprolidine, an HIV drug, is first generation histamine H1 antagonist used in allergic rhinitis (and is clustered with other rhinitis drugs). Cycrimine is a central anticholinergic drug designed to reduce the levels of acetylcholine in the treatment of Parkinson's disease, while its neighbor Carbinoxamine, used for allergic rhinitis, is likely capable of treating mild cases of Parkinson's disease as well (http://www.ebi.ac.uk/chebi/searchId.do?chebiId=3398). It should be noted that since SLAP does not differentiate positive and negative interactions (activation or inhibition), the pairs may present opposite indication. Phenylpropanolamine (an Alpha-1A adrenergic receptor agonist), clustered with Doxazosin (an Alpha-1A adrenergic receptor antagonist for treating hypertension) is known to cause severe hypertension [36].

## Discussion

In this paper we demonstrate the SLAP method of association prediction and the utility of predicting associations based on semantic networks. The method performs extremely well in correctly identifying known drug-target pairs in the data, has been shown to outperform similar link prediction methods used in social networking, and compares favorably with the established SEA method for predicting new drug-target interactions, as well as with the CMap method for associating drugs with changes in gene expression levels. We introduce the use of a drug-similarity network based on association profiles of drugs across targets, and use these to propose potential new drug indications, although these indications have not yet been validated experimentally.

The use of large semantically annotated datasets to identify potential relationships from the linked data is a very new area, and we consider this an initial work in this field. There are several limitations to our current version. First, adding more data pertaining to drugs and targets would help identify more pairs. The side effect, disease and chemical ontology data are only linked to a limited number of drugs at present, and protein-protein interaction and protein pathway mapping data should greatly enhance its utility. In particular, the ability to embed compounds into the network for which there is no public information using chemical structure similarity, or new targets into the network using sequence similarity, would enable predictions to be made (albeit more indirectly) for newly synthesized or resolved compounds and targets. Second, as the complexity of path finding increases dramatically with increasing path length, only shortest paths with length 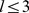 was considered, thus potentially missing important path patterns that have a greater path length. Third, edge weights are defined with the assumption that the probability from one node to its neighbors with same semantic type (e.g., from one drug to its targets) is equal. An important limitation of our current algorithm is that it does not enable differentiation of relationships other than categorical ones defined in the ontology. For instance, binding affinity could be used to weight the edge between drug and target, the edge with lower affinity is expected to have higher probability than that with higher affinity (or inactive interaction). Using such data brings up the issue of comparability between datasets: some chemogenomics datasets such as DrugBank currently do not provide sufficient binding affinities, but the weighting schema can be modified straightforwardly in SLAP once the data is provided. In addition, binding types (agonist/antagonist, activator/inhibitor) can be incorporated to classify and weight edges. Fourth, it should be pointed out that using large public integrated datasets means there is often a fuzziness between “no data” and “inactive data”: i.e. we cannot assume that because two items do not have a relationship in the dataset, that they are not related - for instance that a drug cannot inhibit a target.

A key question in employing any drug-target prediction method is the extent to which it requires data completeness - in the extreme a full experimental matrix - to work properly (i.e. if it needs to be trained with consistent known active/inactive information for all compounds against all targets). Our methods does not require such training, indeed its purpose is to suggest potential “missing links” in incomplete data. However, it should be pointed out that the level of data completeness in a set will affect the path lengths, z-scores and associations scores produced. We believe that overall SLAP should be considered a useful tool for predicting that a relationship exists between drugs and targets, and thus as a tool primarily for ideas generation and for suggesting relationships to be probed experimentally: its purpose is to predict a relationship, not necessarily indicating a strong physical interaction. We believe it is also useful, as demonstrated in our drug network, for profiling compounds by their target associations (and vice versa) and we plan to explore other types of network that can be derived from SLAP.

Many drug target prediction methods only employ single kinds of information or relationship (e.g., substructure, side effect, etc.), these methods are limited due to incompleteness of the data, for instance drug target relation are far from complete [37]. The employment of various data information can compensate for the lack of completeness of individual information. SLAP shows a direction to leverage such information for drug target prediction. Several sample pairs along with their key information are listed in Table S3. For instance, the association between pyridoxal phosphate (CID: 1051) and cysteine conjugate-beta lyase 2 (CCBL2) is very strong (p-value = 1.9E-3), but if we removed gene ontology information, their association would become very weak (p-value = 0.02); the association between Dexamethasone (CID:5743) and annexin A1(ANXA1) would hardly be captured if substructure information were not considered.

The most compelling advantage of SLAP is its consideration of relations from a system level rather than just by known binding affinity data. Other than direct drug target interactions, SLAP is also capable of recognizing indirect interactions (e.g., the change of gene expression level) from random pairs, although the association scores are often smaller than direct interactions (Figure S3). It thus allows us to evaluate drug similarity based on the biological function. The network demonstrates that such similarity measurements not only is able to identify the drug action modes but also could suggest the new use of drugs.

## Materials and Methods

### Network building

We extracted drug-target interactions and the data contributing to either the similarity of compounds, the similarity of targets or chemical target interaction from the Chem2Bio2RDF set [24], and added semantic annotations using the Chem2Bio2OWL ontology [31], to create a semantic drug-target network. For example, two compounds are similar if they share same side effects, same substructures or same chemical ontology terms; two targets are similar if they share the same gene ontology terms or ligands, or they function in the same pathway. Ten classes of entities and 12 link types were defined in Table S1 and Table S2 respectively. A link between a drug and a target via bind type is established if there is a binding affinity smaller than 30 um if exists. Each node in the network is an instance of one of the classes. The detailed information on the collection of individual nodes and edges are in the supporting Text S1.

### Drug target pairs preparation

Drug target pairs from DrugBank were used to build the network. We took only the pairs in which drugs were small molecules (by mapping to PubChem) and targets are Homo sapiens (by mapping to HGNC). A total of 5607 pairs were extracted from the network as one benchmark dataset for model evaluation. The drug target pairs were grouped into 6 classes according to ChEMBL [38] target classification (i.e., enzyme (2393 pairs), membrane receptor(862 pairs), ion channel(392 pairs), transporter(209 pairs), transcription factor (208 pairs) and others (1543 pairs)). Another benchmark dataset was created from MATADOR [35] which was not used for network building. We took drug target pairs with direct interaction types and confidence score 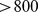 from MATADOR. 1176 direct pairs in MATADOR were used, in which 1065 pairs have at least one path with length 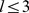. 3665 indirect pairs in MATADOR were also extracted for evaluating indirect drug target interaction. Indirect interactions are caused by many different mechanisms, such as binding with drug metabolites or changing gene expressions [35].

### Path finding

A heap-based Dijkstra algorithm was employed to quickly find the paths between two nodes [39], [40]. It can achieve a complexity of O(nlogn). Each path is represented as: 
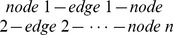
. The length of a path is the number of edges between two nodes. We only took the paths of length 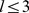. Only significant paths (assessed by statistical models) are visualized in Cytoscape [41].

### Path association

Let graph as 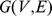, 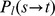 as the 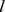
th shortest path from node 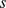 to 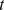. 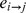 as the edge from node 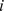 to node 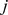. 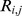 as the link (relation) type of 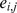.

It is assumed that it has an equal probability traversing node 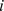 to its neighbor node 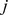 within the same type, thus:
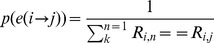
where 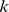 is the degree of node 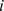.

As the probability of each edge is independent, the probability traversing from 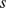 to 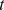 via a path is:
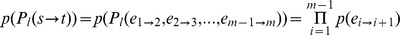
where m is the number of nodes in the path. Since p is very small, the logarithm is applied,
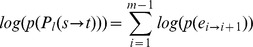



Accordingly, the probability traversing from 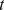 to 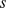 via a path is:
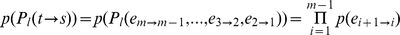


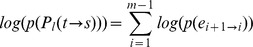



We consider the graph as undirected, then we take the average as the raw score of path 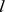 between 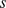 and 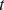:
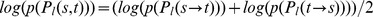



### Statistical model

We randomly sampled 100,000 drug target pairs from DrugBank covering 1355 approved small molecular drugs and 1683 human targets, 54,414 pairs have at least one shortest path with length 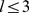. The sampling yielded 2,344,026 paths, which were categorized into 34 path patterns. The scores of each pattern were fitted to a normal distribution (Figure S1) and the expected mean and standard deviation were estimated, followed by calculation of the z score of every path. Only the paths with z score greater than 0 were considered as the valid paths contributing to the association. The z scores of all the valid paths from 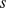 to 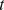 were summed up to get its association score, which was later used to measure the strength of the association.
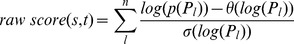
where 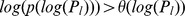; n is the number of shortest paths between the nodes 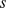 and 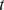; 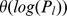 and 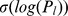 are expected mean, expected standard deviation of the pattern to which 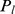 belongs.

Some patterns may be not helpful or even noisy for assessing drug target association. We built a test set consisting of drug target pairs from DrugBank and the same number of random drug target pairs sampled from the set of drugs and targets composing the real drug target pairs. For one pair, raw scores of all the paths within a path pattern were calculated and summed up as a score for that path pattern. The scores were then used to rank the pairs in the test set. The evaluation of each pattern was performed using the area under ROC. We also applied the same procedure to the direct pairs from MATADOR. The patterns with low ROC (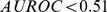) were considered as uninformative. The uninformative patterns agreed by both test sets taken from DrugBank and MATADOR were removed.

The logarithmic association scores of random pairs conforms to a normal distribution (Figure S2); p-value is estimated to show the probability of observing a given score by random chance alone. Lower p-value indicates stronger relation between two objects.

### Model evaluation

A test set was composed of a set of drug target pairs from DrugBank and the same number of random pairs as decoys. Three another test sets were created by increasing the number of random pairs such that the sizes of random pairs are 4, 8 and 12 times more than true drug target pairs. For each pair, the paths including the direct link if exists were removed, and the z scores of all valid paths were summed up as association score. The scores were ranked to generate ROC curves [42], which are widely adopted to measure drug target prediction methods [20], [22], [33], [43]. We also considered Precision and Recall (PR) curve, which shows the ratio of true positives among all the predicted positives under a given recall rate [44]. PR curve is more informative and biologically meaningful while the dataset is imbalanced. The same procedure was also applied to another dataset collected from MATADOR. Other than using SLAP scores, we considered the number of shortest paths (maximum length 3), the number of valid paths (significant path defined in the model), the sum of raw score of all paths, the max raw score among all paths, and the average raw score of all paths. In addition, we took the pairs validated in experiments in a recent published paper [7] as novel pairs, after manually mapping their drugs and targets to PubChem CIDs and gene symbols, we ran SLAP to get p-values of all the valid pairs.

### Assess drug similarity

We identified drug-disease pairs from Yildirim et al. [45], then mapped the drugs to PubChem CIDs (the default compound identifier in the network). Many drugs have multiple indications, so in order to visualize drugs by therapeutic indications, only drugs with one indication were kept. We also only kept the top 10 diseases ordered by the number of related drugs. The association scores of all mapped drugs against a set of human targets construct biological signatures which were later used for measuring drug similarity using Pearson correlation coefficient. The pairs with coefficient 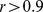 constitute the network. Drug structural similarity was measured by Tanimoto coefficient using MACCS fingerprint.

## Supporting Information

Figure S1 Raw score distribution of 8 path patterns.
(TIFF)

Figure S2 Fit association scores of random pairs to a normal distribution. Logarithm is applied to the scores. R2 is 0.96.
(TIF)

Figure S3 Logarithmic association scores of direct drug target pairs versus indirect pairs. Indirect pairs were taken from MATADOR.
(TIF)

Figure S4 Logarithm association scores of pairs among five gene families and random pairs.
(TIF)

Figure S5 Precision and Recall curve under different ratios between the number of true drug target pairs and the number of random drug target pairs. (a) ratio = 1∶1 (b) ratio = 1∶4 (c) ratio = 1∶8 (d) ratio = 1∶12.
(TIFF)

Figure S6 (a) Sample Insomnia related drugs (b) Levodopa vs Methyldopa.
(TIFF)

Table S1 Node type information.
(DOCX)

Table S2 Edge type information.
(DOCX)

Table S3 Sample drug target pairs with/without key information contributing to the association.
(DOCX)

Table S4 Comparing with SEA.
(XLSX)

Table S5 Comparing with CMap.
(XLSX)

Text S1 Dataset preparation.
(DOCX)
