# Supplementary figures and images for: Assessing Drug Target Association Using Semantic Linked Data
Source: PLoS Comput Biol. 2012 Jul 5;8(7):e1002574. doi: 10.1371/journal.pcbi.1002574 (PMC3390390; doi:10.1371/journal.pcbi.1002574)

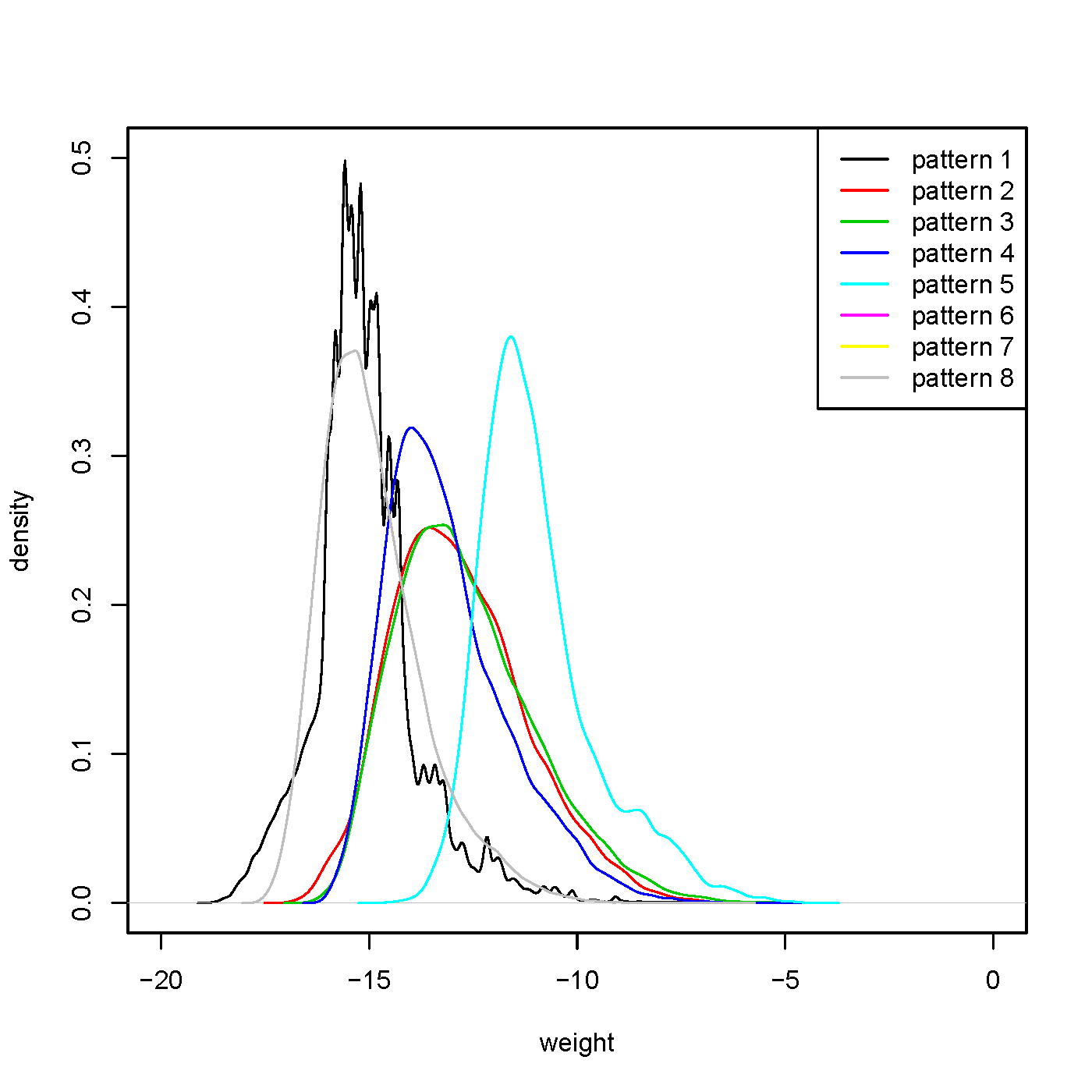

Supplement: Figure S1 — Raw score distribution of 8 path patterns. (TIFF) [file pcbi.1002574.s001.tif]

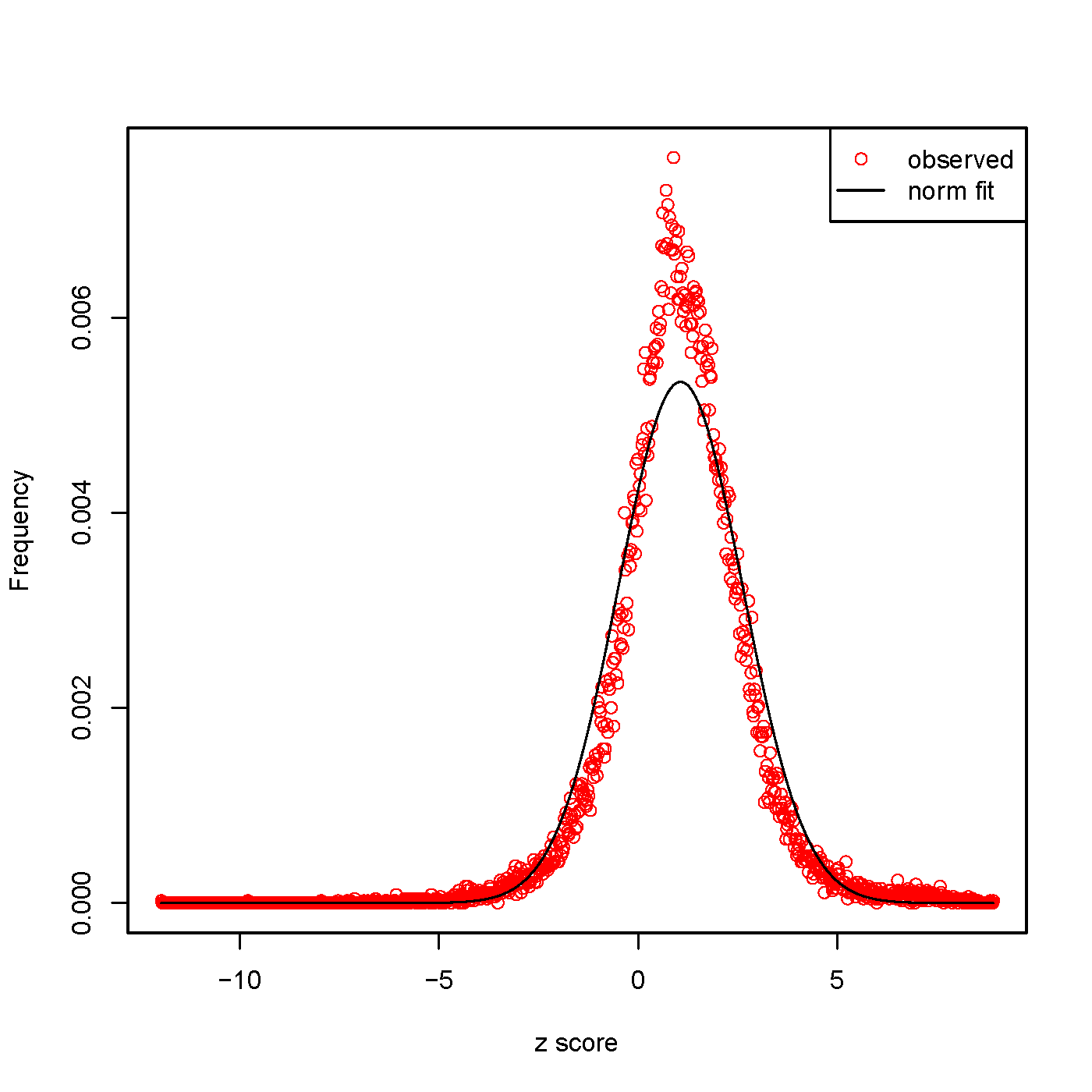

Supplement: Figure S2 — Fit association scores of random pairs to a normal distribution. Logarithm is applied to the scores. R2 is 0.96. (TIF) [file pcbi.1002574.s002.tif]

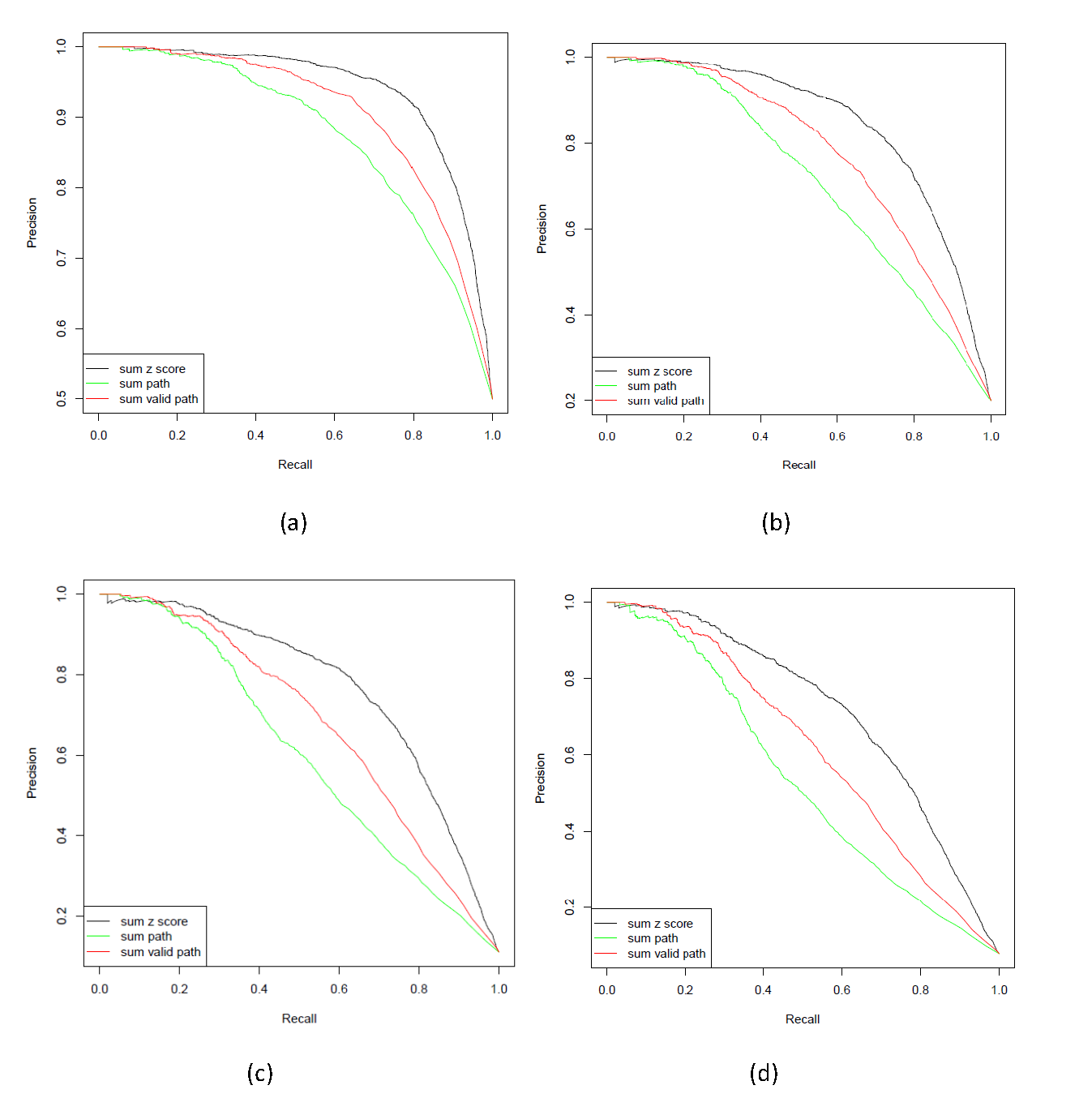

Supplement: Figure S5 — Precision and Recall curve under different ratios between the number of true drug target pairs and the number of random drug target pairs. (a) ratio = 1∶1 (b) ratio = 1∶4 (c) ratio = 1∶8 (d) ratio = 1∶12. (TIFF) [file pcbi.1002574.s005.tif]

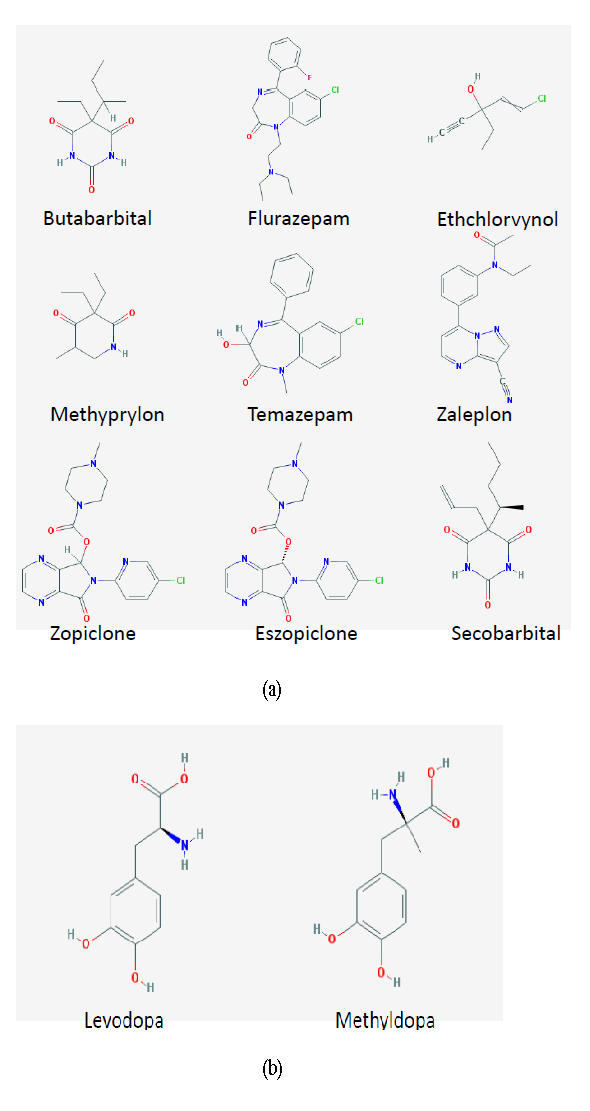

Supplement: Figure S6 — (a) Sample Insomnia related drugs (b) Levodopa vs Methyldopa. (TIFF) [file pcbi.1002574.s006.tif]
