# Supplementary material for: Assessing Drug Target Association Using Semantic Linked Data
Source: PLoS Comput Biol. 2012 Jul 5;8(7):e1002574. doi: 10.1371/journal.pcbi.1002574 (PMC3390390; doi:10.1371/journal.pcbi.1002574)
Supplement: Table S1 — Node type information. (DOCX) [file pcbi.1002574.s007.docx]

Table S1: Node type information

| Classes | description | data sources | # instances | identifier |
| --- | --- | --- | --- | --- |
| Chemical Compound/Drug | a small bioactive molecule | PubChem, DrugBank | 258033 | PubChem CID |
| Target | gene or gene expression products | UNIPROT | 21733 | Gene Symbol |
| Pathway | a set or series of biological interactions | KEGG | 192 | KEGG ID |
| Disease | a disordered or incorrectly functioning organ of body | OMIM | 1284 | OMIM ID |
| Side effect | undesired effect from a medicine | SIDER | 1051 | UMLS ID |
| Tissue | an ensemble of cells | HPRD | 507 | Tissue Name |
| Gene family | HGNC gene family | HGNC | 329 | Gene family name |
| Gene ontology | gene ontology term | GOA | 9710 | GO ID |
| Chemical ontology | chemical ontology term | ChEBI | 2777 | ChEBI ID |
| Substructure | Chemical substructure | DrugBank | 290 | Substructure Name |
