# Supplementary material for: Assessing Drug Target Association Using Semantic Linked Data
Source: PLoS Comput Biol. 2012 Jul 5;8(7):e1002574. doi: 10.1371/journal.pcbi.1002574 (PMC3390390; doi:10.1371/journal.pcbi.1002574)
Supplement: Table S2 — Edge type information. (DOCX) [file pcbi.1002574.s008.docx]

Table S2: Edge type information

| node 1 | relation | node 2 | data sources | edges | node1 | node2 |
| --- | --- | --- | --- | --- | --- | --- |
| Chemical Compound/Drug | hasSubstructure | Substructure | DrugBank | 6169 | 1313 | 290 |
| Chemical Compound/Drug | hasChemicalOntology | Chemical Ontology | ChEBI | 15986 | 899 | 2777 |
| Chemical Compound/Drug | bind^*^ | Target | ChEMBL,BindingDB,PubChem BioAssay, TTD, DrugBank, DPSP, KEGG | 522543 | 256752 | 4726 |
| Chemical Compound/Drug | express | Target | CTD | 16146 | 1525 | 4343 |
| Chemical Compound/Drug | treatDisease | Disease | Diseasome | 943 | 572 | 189 |
| Chemical Compound/Drug | causeSideEffect | Side effect | Sider | 9004 | 789 | 1051 |
| Target | proteinProteinInteraction | Target | HPRD | 30215 | 7303 | 6777 |
| Target | hasPathway | Pathway | KEGG | 11258 | 3857 | 192 |
| Target | hasGeneFamily | Gene family | HGNC | 7181 | 7181 | 329 |
| Target | hasGO | Gene ontology | UniProtKB-GOA | 95419 | 14483 | 9710 |
| Target | causeDisease | Disease | OMIM | 2929 | 1778 | 1284 |
| Target | expressIn | Tissue | HPRD | 10178 | 3971 | 507 |
| *bind: IC50 or EC50 < 30um if exists. | | | | | | |
