# Supplementary material for: Assessing Drug Target Association Using Semantic Linked Data
Source: PLoS Comput Biol. 2012 Jul 5;8(7):e1002574. doi: 10.1371/journal.pcbi.1002574 (PMC3390390; doi:10.1371/journal.pcbi.1002574)
Supplement: Table S3 — Sample drug target pairs with/without key information contributing to the association. (DOCX) [file pcbi.1002574.s009.docx]

Table S3: Sample drug target pairs with/without key information contributing to the association.

| CID | Drug name | Gene symbol | Target name | Key information | p value without key information | p value with key information |
| --- | --- | --- | --- | --- | --- | --- |
| 6741 | Methylprednisolone | NR3C1 | glucocorticoid receptor | Chemical Ontology, Expression | 0.03 | 8.0E-4 |
| 5743 | Dexamethasone | ANXA1 | annexin A1 | substructure | 0.015 | 3.8E-3 |
| 8223 | Ergotamine | ADRA1A | adrenergic, alpha-1A-, receptor | ligand | 0.09 | 4.0E-5 |
| 1051 | Pyridoxal Phosphate | CCBL2 | cysteine conjugate-beta lyase 2 | Gene ontology | 0.02 | 1.9E-3 |
