# Supplementary material for: Assessing Drug Target Association Using Semantic Linked Data
Source: PLoS Comput Biol. 2012 Jul 5;8(7):e1002574. doi: 10.1371/journal.pcbi.1002574 (PMC3390390; doi:10.1371/journal.pcbi.1002574)
Supplement: Text S1 — Dataset preparation. (DOCX) [file pcbi.1002574.s012.docx]

# Supporting materials

## Datasets

Many of the relevant data sources are scattered around the web and published in very diverse formats (text files, journal articles, XML, relational databases, and so on) and may be structured or unstructured. Some of them are homogeneous but with slightly different foci. The semantic relationship of these datasets to each other is often unclear. Semantic Web techniques offer an efficient way to annotate data and integrate them into a huge network. The network is presented as a list of triples, which consist of subjects, predicates and objects. Subjects and objects are presented as nodes and predicates are presented as edges in the network. Each entity has a URI, a universal resource identifier. For example, compound Troglitazone is presented as http://chem2bio2rdf.org/pubchem/resource/pubchem_compound/5591. Each individual instance is mapped to a class. The major classes are listed in table S1; they are linked by object property (table S2). There may be different types of edges linking two nodes. Compound could link to Target by either binding or the affection of expression.

In data integration, all data have a unique agreed identifier (table S1) and other identifiers were mapped to the unique identifier by in house scripts and manual inspection. The general integration procedure was explained in our previous works(Chen, Dong et al.; Chen, Ding et al.; Chen, Ding, Wild). Some changes for this particular case are pointed out here. We used expression data from CTD (by searching the interaction type =`expression') for express type. The binding type requires the activity is less than 30um if exists. GO terms were filtered out by using qualifier!='NOT' and evidence!='NAS' and evidence!='ND' and evidence!='NR'(Rhee, Wood et al.). We noticed that most of the edge types (e.g, protein protein interaction, drug target binding, etc) conform to a scale free property, in which degree distribution follows a power law, but some promiscuous nodes (with many neighbors) skewed the distribution, to name a few: GO:0005515 (molecular function) in GO class; CHEBI:25700 (organic molecular entity ) in Chemical Ontology Class; C0027497(Nausea) in Side Effect Class, Aromatic compounds in Substructure class, Brain in Tissue classes. These high promiscuous nodes were removed manually from the network. The whole dataset is available at the website (<http://chem2bio2rdf/slap>).

## References

Chen, B., X. Dong, et al. "Chem2Bio2RDF: a semantic framework for linking and data mining chemogenomic and systems chemical biology data." Bmc Bioinformatics **11**: 255.

Rhee, S. Y., V. Wood, et al. (2008). "Use and misuse of the gene ontology annotations." Nat Rev Genet **9**(7): 509-15.

Chen, B, Y. Ding, et al. "Chem2Bio2RDF: A Linked Open Data Portal for Systems Chemical Biology" Web Intelligence and Intelligent Agent Technology (WI-IAT), Toronto, 2010

Chen, B., Y. Ding and DJ Wild. Improving integrative searching of Systems Chemical Biology data using semantic annotation, Journal of Cheminformatics, 2012, 4:6
